# Social connection interventions and depression in young adults: a systematic review and meta-analysis

**DOI:** 10.1007/s00127-024-02722-1

**Published:** 2024-08-16

**Authors:** Clotilde Vazquez Alvarez, Luwaiza Mirza, Jayati Das-Munshi, Tassia Kate Oswald

**Affiliations:** 1 Department of Psychological Medicine, Institute of Psychiatry, Psychology & Neuroscience, King’s College London, London, UK; 2 University Hospitals Sussex, Sussex, UK; 3 ESRC Centre for Society and Mental Health, King’s College London, London, UK; 4 South London & Maudsley NHS Trust, London, UK; 5 Population Health Improvement UK (PHI-UK), London, UK

**Keywords:** Depression, Social connection, Loneliness, Intervention, Young adults, Emerging adulthood

## Abstract

**Purpose:**

Early adulthood is a period which may increase vulnerability to loneliness and mental health difficulties among young adults. Social networks play an important role in buffering against adverse mental health, but there is a lack of evidence around whether social connection interventions could play a role in preventing mental health difficulties for young adults.

**Methods:**

A systematic review and meta-analysis was conducted (PROSPERO ID: CRD42023395595). PubMed, PsycInfo, and Scopus were searched (01 January 2000–01 January 2023). Studies were eligible if they (i) were quantitative, (ii) included young adults (18–24 years) from the general population, (iii) tested a social intervention which aimed to increase the quantity or quality of social connections or reduce loneliness, (iv) had a comparison group, and (v) measured depression and loneliness/social connection as outcomes. Following study screening and selection, the data extraction and risk of bias assessments were independently conducted in duplicate. The Cochrane RoB-2 tool and ROBINS-I tool were used to assess risk of bias. Results were narratively synthesised and random effects meta-analysis with standardised mean differences was conducted.

**Results:**

Six studies were included; four in-person interventions with higher education students, one online intervention with higher education students, and one intervention for youth involved in street life. The studies were mostly rated as having some or moderate concerns with risk of bias. The interventions were associated with an overall mean reduction in depression for young adults (SMD = -0.19; 95% CI, -0.33 to -0.05; *p* = 0.008; 4 studies, excluding studies with serious risk of bias). All interventions had beneficial effects on a range of diverse social connection outcomes, but there was no overall statistically significant mean reduction in loneliness for young adults in pooled analyses (SMD = -0.10; 95% CI, -0.24 to 0.05; *p* = 0.188; 3 studies).

**Conclusion:**

Social connection interventions show some promise in improving depression and social connection outcomes in young adults but more high-quality research, across diverse settings, is needed in this area.

**Supplementary Information:**

The online version contains supplementary material available at 10.1007/s00127-024-02722-1.

## Introduction

Mental disorders are the leading cause of overall disease burden for young people worldwide [1]. Three quarters of mental health problems emerge before 25 years of age [2], and there is evidence that mental disorders are becoming more prevalent in young people [3, 4]. Depression is a common mental disorder which has a range of impacts on individuals, as well as their families and communities. Health-related impacts of depression include suicide, physical illness, and premature mortality, while non-health related outcomes include poorer education and employment outcomes [5].

It is widely documented that loneliness is associated with an increased risk for depression [6, 7]. For example, recent research indicates that measures of loneliness in young adults are predictive of future antidepressant use [8]. Loneliness is defined as a discrepancy between the quantity and quality of desired social relationships and actual social relationships [8], and has recently been recognised as a major public health concern for societies [9].

Research indicates that experiences of loneliness are highest in younger adults and older people [10, 11]. A recent cross-temporal meta-analysis of 437 independent samples of young adults indicated that there have been increases in loneliness since 1976, implying that loneliness is a rising concern among young adults [12]. More recently, data shows that young adults experienced greater feelings of loneliness and depression during the COVID-19 pandemic than other age groups. These findings were seen across numerous high-income countries [7, 13–16], with a US study reporting that increases in loneliness accounted for much of the increase in depression among young adults during the pandemic [17].

Early adulthood is a unique developmental period characterised by social, environmental, educational, economic, cognitive, and psychological change [8]. Contemporary cohorts of young adults have also grown up alongside significant societal developments which have changed the nature of human relationships, such as greater mobility opportunities and changes in communication due to technological advancements [12]. The culmination of these unique experiences may explain young adults’ vulnerability to both loneliness and mental health difficulties.

Given the relationship between loneliness and depression, social interventions which aim to increase social connections, or reduce loneliness, may be important in protecting against depression in young adults. This is further supported by the claim that social capital, as a major social determinant of mental health, is protective against depression [18, 19]. Most literature concerning social connection interventions currently focuses on children and adolescents [20–22], older people [23], or clinical samples with pre-existing mental health problems [24–26] only. A lack of focus on social connection interventions for young adults in the general population is apparent. This was recently reflected by a 24-year-old participant in a qualitative study, in which she described early adulthood as *“a time in life that often gets overlooked”* [27].

It is important to address this gap given the unique challenges presented in early adulthood and current prevalence of mental health problems and loneliness. Systematically reviewing the effectiveness of social connection interventions in reducing depression among young adults is necessary to address this gap. The aims of the current review were to (1) identify and present interventions which address social connection/loneliness in young adults, and (2) describe the effectiveness of these interventions in changing depression and social connection/loneliness outcomes.

### Methods

A systematic review was conducted, following the Preferred Reporting Items for Systematic Reviews and Meta-Analyses (PRISMA) checklist (Supplementary File 1). A protocol was pre-registered on PROSPERO (CRD42023395595). In the protocol, it was originally anticipated that heterogeneity in outcome measures would mean that meta-analysis would not be possible. Following study selection and data extraction, it was evident that meta-analysis was possible for depression and loneliness outcomes and was therefore added to the review.

### Data source

Three databases were searched: PubMed, Scopus, and PsycInfo. The searches were conducted from 01 January 2000 to 01 January 2023 and were limited to English language. The search strategy included terms such as: “young adults”, “social interventions”, “social connectedness”, “depression”, and “randomized controlled trials” (see Supplementary File 2 for the full search strategies).

### Inclusion/exclusion criteria

#### Study designs

Peer-reviewed quantitative studies assessing the effectiveness of an eligible intervention were included. This included randomised controlled trials (RCTs), quasi-experiments, and pre-post studies with a comparison group. Qualitative studies, reviews, commentaries, editorials, and book chapters were excluded.

#### Participants

Young adults aged 18–24 years were included, and all other age groups were excluded. Participants from any setting (e.g., educational, occupational, community, others) in the general population were eligible for inclusion. Clinical patient populations explicitly recruited from clinical settings like mental health services were excluded.

#### Interventions

Interventions aiming to increase the quantity or quality of social connections or reduce loneliness in young adults in the general population, were included. These interventions could be in-person or online but needed to explicitly state that they aimed to increase social connections or reduce loneliness. For example, social media platforms were not automatically included as “social interventions” because, while some social media platforms (e.g., Facebook, Instagram) may have initially been designed with the intention to socially connect users through two-way interaction, they have evolved over time to become marketing tools with in-built designs that commonly encourage one-way non-interactive engagement (e.g., scrolling a social media feed with products, advertisements, and entertainment) [28].

Interventions which exclusively used psychological approaches such as cognitive behavioural therapy (CBT), mindfulness, or acceptance and commitment therapy (ACT), were excluded.

#### Comparator

Studies had to include a comparison group. Eligible interventions could be compared to either no intervention or to a different intervention.

#### Outcomes

To be eligible for inclusion, studies had to include both our primary and secondary outcomes of interest. The primary outcome we assessed was the effect of interventions on changes in the severity, course, or prevalence of depression or depressive symptoms. Studies needed to have a measure of depression at baseline and after the intervention, using a valid and reliable tool. Secondary outcomes we assessed were measures of social connection(s) and/or loneliness, which also had to be measured using a valid and reliable scale.

### Context/settings

No restrictions were placed on the context/settings. Studies were included from low-, middle-, and high-income countries. Interventions could be conducted in a range of settings, such as college/university, sporting clubs, or in community settings more widely.

### Study screening and selection

The electronic database search results were downloaded to Endnote 20.4.1. All titles and abstracts were screened by one reviewer (CVA). Applying the inclusion criteria, studies were either excluded or progressed to a full-text screening stage if they were potentially relevant. One reviewer screened all full-text articles (CVA), and a random 10% sub-sample were screened by a second reviewer (TKO). The two reviewers had 100% agreement. The reference lists of the included articles were also screened to ensure no studies were missed. The corresponding author of each included article was contacted to request any additional studies meeting the eligibility criteria.

### Data extraction

Data extraction was conducted in Microsoft Excel, using a form designed and tested by the study authors. Data extracted included: first author, year of publication, study design, location, description of population (including age, gender, ethnicity), description of intervention, intervention delivery mode (online or in-person), total number of participants (at baseline and at follow-up), depression measure used, social connection/loneliness measure used, main findings related to depression, main findings related to social connection/loneliness outcome, and funding information. All data was independently extracted by two authors (CVA and LM) and discrepancies were addressed by a third author (TKO).

### Risk of bias assessments

Risk of bias assessments were conducted using two tools. The Cochrane Risk of Bias tool for Randomised Controlled Trials (RoB 2.0) was used for studies that used a randomised controlled trial design. The Risk of Bias in Non-Randomised Studies (ROBINS-I) tool was used for studies that used a non-randomised design. All studies were independently assessed by two reviewers (CVA and LM). Any discrepancies between the reviewers were resolved through discussion, and the other reviewers (TKO and JDM) were consulted to resolve final uncertainties.

### Narrative synthesis

Narrative synthesis was conducted and summaries of the included interventions, depression outcomes, and social connection/loneliness outcomes were tabulated. To compare intervention types, studies were grouped by their intervention delivery modality (e.g., in-person or online) and by the study sample type (e.g., higher education students or not).

### Statistical analysis

Random effects meta-analyses were performed, utilising standardised mean differences (SMD) to account for different depression and loneliness measures used across studies, with an assessment of I^2^ for heterogeneity. The *metan* suite of commands were utilised in STATA/MP 18.0 for the analysis. To assess for publication or small study biases, we created and visually inspected a funnel plot. Egger’s test was not used as there were less than 10 studies [29]. We presented overall effects as well as subgroup effects for the different intervention types, separated by delivery modality and study sample type. Sensitivity analyses were also conducted and reported by excluding the studies which were assessed as having serious risk of bias.

## Results

**Fig. 1:**
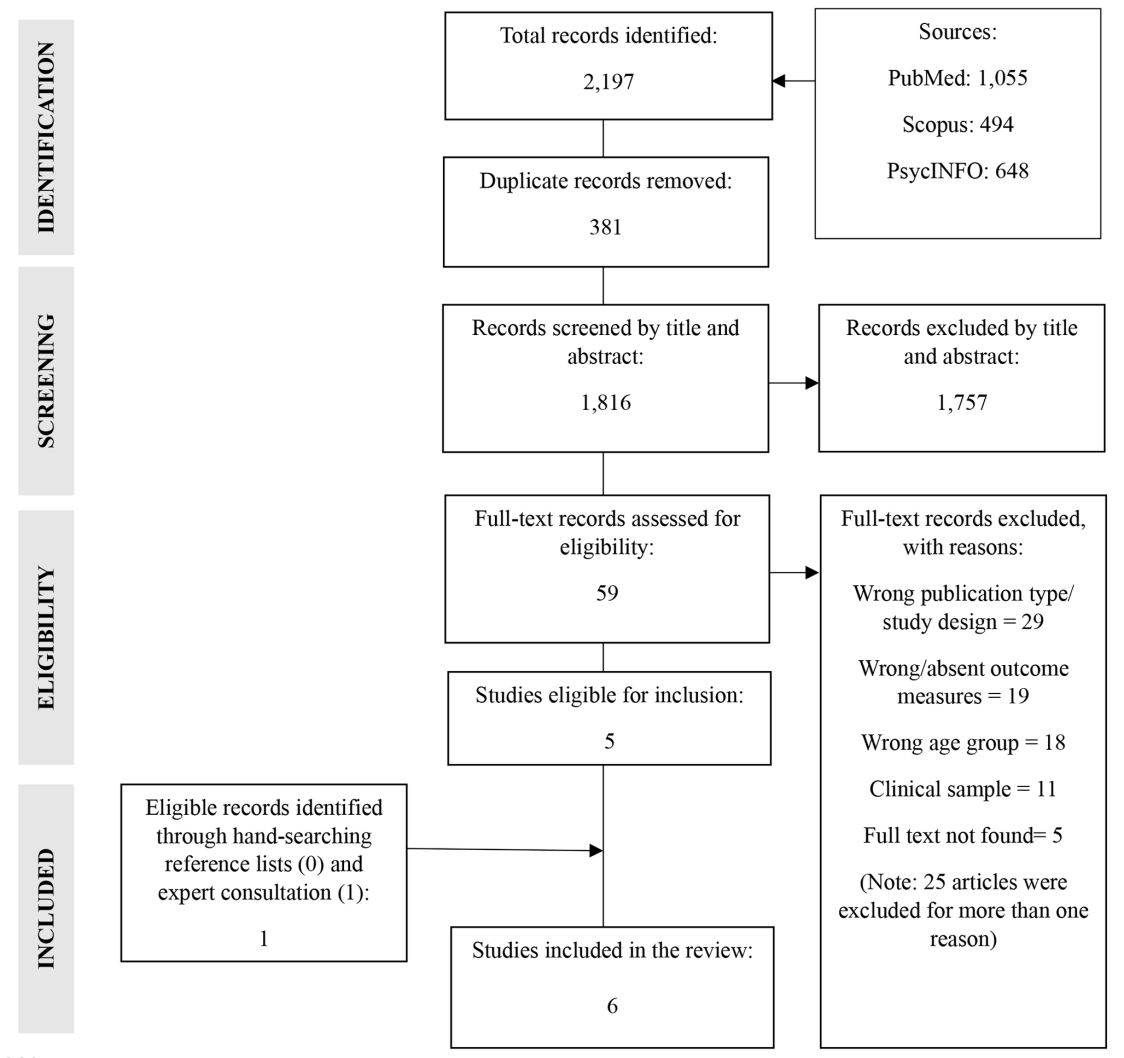
PRISMA flow diagram for study identification, screening, and selection

The study search, screening, and selection process is presented in Fig. 1. A total of 2,197 articles were retrieved, of which 1,055 were from PubMed, 494 from PsycInfo, and 648 from Scopus. After duplicates were removed (*n* = 381), articles were excluded based on the title and abstract (*n* = 1,757). A final 59 articles were screened at the full-text stage. Of those, only five met the inclusion criteria. Reasons for exclusion of full-text articles are indicated in Fig. 1 and in Supplementary File 3. No additional studies were identified through hand-searching the reference lists of included papers. One additional eligible study was obtained through contacting the corresponding authors of included papers. Six studies were included in the final systematic review and meta-analysis.

### General study characteristics

The general study characteristics of the six included studies are presented in Table 1. Three studies employed randomised controlled designs [30–32] and the remaining three utilised non-randomised designs. Three studies were conducted in the USA [30–32], and the others in Canada [33], Australia [34], and South Korea [35]. Sample sizes ranged from 23 to 438 participants at baseline. Mean participant ages ranged from 18.68 to 21.56 years.

**Table 1:** General study characteristics

		Brady et al., 2020	Bruehlman-Senecal et al., 2020	Costello et al., 2022	Haslam et al., 2016	McCay et al., 2011	Yoon et al., 2011
**Study design**		Randomised controlled trial (follow-up from Walton et al., 2011 [36])	Pilot randomised controlled trial	Randomised controlled trial	Pilot non-randomised controlled study	Non-randomised pilot study	Non-randomised study
**Location**		California, USA	Northwestern USA	Virginia, USA	Brisbane, Australia	Toronto, Canada	Seoul, South Korea
**Sample** Baseline; Follow-up; Attrition (%)		92; 80; 13%	221; 209; 5.4%	438; 369; 15.8%	158; 51; 67.7%	23; 15; 34.8%	70; 64; 8.6%
**Sample characteristics**	*Description*	First year students at a well-resourced USA college, followed up post-college.	First year students from a University in northwestern USA.	First year and transfer students from a south-eastern University.	Undergraduate University students who reported isolation and moderate distress.	Youth from community agencies that provide services to youth involved in street life.	Nursing students across three universities.
	*Mean age (SD)*	During intervention: First year students At 8.5-year follow-up: 27.42 (1.31) years	18.68 (0.35) years	18.98 (1.03) years	Intervention: 20.95 (5.05) years Control: 20.20 (2.48) years	Intervention: 21.56 (2.70) years Control: 21.00 (2.45) years	Range: 19–29 years Intervention: 80.6% aged 19–20 years Control: 72.7% aged 19–20 years
	*Gender %*	Intervention: 62% female Control: 64% female	Female: 59.3% Male: 36.7% Non-binary: 4.1%	Female: 68.9% Male: 30.1% Other: 1%	Intervention: 65.4% female Control: 76% female	Female: 40% Male: 60%	Not stated
**Intervention**	*Description*	*Social Belonging Intervention*, a one-hour immersive experience in which participants read the results of a survey of older college students, in which social belonging challenges were presented as a normal and temporary part of the transition to college, which was common across ethnic and gender groups. Participants were asked to write a reflective essay about their own experiences and present it as a speech to a video camera.	*Nod*, a mobile app which incorporates positive psychology, mindfulness-based self-compassion, and CBT skill building exercises to address loneliness among first year college students. *Nod* delivers skills via social challenges (suggested ideas for reaching out to others and taking action to build social connections), reflection activities, and written student testimonials that encourage social connection building.	*The Connection Project* (College Version), a group-based intervention for enhancing belongingness by providing graduated experience of open, authentic, and supportive conversations among the group members.	*Groups 4 Health (G4H)*, a group-based intervention for social identity and connection. Modules contain a series of exercises and discussions that target development and maintenance of social group relationships.	*Relationship-based program*, focussing on relationships that would guide, support, and nurture youth. Sessions focussed on social support and networks, positive self-concepts and resilience, emotional understanding, self-determination, and choice.	*Park’s interpersonal relationship program*, aims to improve university students’ interpersonal relationships by finding balance and harmony in one’s cognition, emotion, behaviour, and interaction with other people.
	*Delivery*	In-person	Online app-based intervention	Hybrid intervention, originally designed and delivered in-person and then online after the COVID-19 pandemic.	In-person	Not stated	In-person
	*Duration*	1 session	4 weeks	9 weeks	2-month intervention period (6-month follow-up)	6 weeks	10 weeks
	*Dosage*	1 h	Self-directed App usage	60–75 min per week.	One 60–75-minute session per week for 4 weeks + one 60–75-minute session one month later.	1.5 h per week.	90 min per week.
**Comparison**	*Description*	Control students participated in a similar one-hour process to the intervention participants, but the process focused on topics unrelated to belonging, such as changes in social-political attitudes.	Waitlist. Access to the app at week 4.	Waitlist. Offered priority access to the program in the following semester.	No intervention.	No intervention.	No intervention.
**Outcome measures**	*Depression*	CESD-10	PHQ-9	BDI	DASS-21	CES-D	CES-D
	*Social connection / loneliness*	Three Item Loneliness Scale; Interpersonal Support Evaluation List	UCLA-8; Perceived Social support; Campus belonging	UCLA Loneliness scale; Sense of school membership scale	UCLA Loneliness scale; Social Adjustment Scale (social functioning)	SCS-R	Interpersonal relationship change scale
**Funding information**		Funding support was provided by the Robert Wood Johnson Foundation and the National Science Foundation.	The study was sponsored by Hopelab Foundation.	This study was supported by the William T. Grant Foundation, the National Institute of Child Health and Human Development, the Jefferson Trust, and the Office of Student Affairs at the University of Virginia.	Funding support was provided by the Canadian Institute for Advanced Research Social Interactions, Identity and Well-Being Program.	Not stated.	Not stated.

***Notes.*** BDI = Beck Depression Inventory; CES-D, CESD-10 = Centre for Epidemiologic Studies Depression Scale; PHQ-9 = Patient Health Questionnaire-9; SCS-R = Social Connectedness Scale-revised; UCLA = University of California, Los Angeles

Five studies recruited higher education students (University or college). Three of these interventions were delivered fully in-person [30, 34, 35], one was delivered completely online via an app [31], and one intervention was hybrid (originally designed and delivered in-person and then partially online due to the COVID-19 pandemic) [32]. One study presented an intervention which focused on youth involved in street life (defined by the original study authors as young people without a home for at least 1 month) and did not report delivery modality [33].

Four of the six studies evaluated group-based social connection interventions [32–35], while one intervention took an individual approach through self-directed app-engagement [31] and another through an individual reading and reflection session [30]. Intervention duration typically ranged from 4-to-10 weeks, and most interventions delivered a weekly session for 60–90 min, with the exception of the intervention which relied on self-directed app engagement [31] and the single session reading and reflection intervention [30].

Five studies included either a waitlist or no intervention control group [31–35], while one study included an active comparison group (not focused on social connection) [30]. Depression was measured using the PHQ-9, BDI, DASS-21, and CES-D. Loneliness was measured in four studies [30–32, 34] and various aspects of social connection were measured across all six studies.

### Risk of bias of included studies

The risk of bias assessments for included RCTs and non-randomised study designs are presented in Tables 2 and 3, respectively. All RCTs were rated as having some concerns with risk of bias overall, mostly due to inability to blind participants to intervention status. Of the non-RCTs, one was rated as having moderate risk of bias, and the remaining two had serious risk of bias. Bias in non-RCTs was mostly due to confounding, deviations from intended interventions, missing data, and in measurement of outcomes.

**Table 2:** Risk of bias assessments for included RCTs

Study	Domain 1	Domain 2	Domain 3	Domain 4	Domain 5	Overall
*Brady et al., 2020*	Some concerns	Some concerns	Low	Low	Low	Some concerns
*Bruehlman-Senecal et al., 2020*	Low	Some concerns	Low	Some concerns	Low	Some concerns
*Costello et al., 2022*	Low	Some concerns	Low	Some concerns	Some concerns	Some concerns

Note: Domain 1 = risk of bias arising from the randomisation process; Domain 2 = risk of bias due to deviations from the intended interventions; Domain 3 = risk of bias due to missing outcome data; Domain 4 = risk of bias in measurement of the outcome; Domain 5 = risk of bias in selection of the reported result

**Table 3:** Risk of bias assessments for included non-randomised studies

Study	Domain 1	Domain 2	Domain 3	Domain 4	Domain 5	Domain 6	Domain 7	Overall
*Haslam et al., 2016*	Moderate	Low	Low	Moderate	Moderate	Moderate	Low	Moderate
*McCay et al., 2011*	Serious	Low	Moderate	Serious	Serious	Moderate	Low	Serious
*Yoon et al., 2011*	Moderate	Low	Low	No information	Moderate	Serious	Low	Serious

Note: Domain 1 = bias due to confounding; Domain 2 = bias in selection of participants into the study; Domain 3 = bias in classification of interventions; Domain 4 = bias due to deviations from intended interventions; Domain 5 = bias due to missing data; Domain 6 = bias in measurement of outcomes; Domain 7 = bias in selection of the reported result

### Main findings

The main findings for depression and social connection / loneliness outcomes are presented in Table 4.

**Table 4:** Main findings for depression and social connection and/or loneliness outcomes

Study	Depression	Social connection and/or loneliness
**In-person interventions with higher education students**		
Brady et al., 2020	At 8.5-year follow-up, there was no statistically significant main effect of intervention on depression scores (B=-0.94; *p* = 0.21; d=-0.29).	At 8.5-year follow-up, there was no statistically significant main effect of intervention on loneliness (B = 0.04; *p* = 0.72, d = 0.08) or social support (B = 0.01, *p* = 0.93, d = 0.02). Black participants experienced increased social support (B = 0.26, *p* = 0.09, d = 0.54), and the race x intervention interaction was statistically significant (B = 0.51, *p* = 0.03).
*Social Belonging Intervention*		
Risk of bias: Some concerns		
Costello et al., 2022	There was a statistically significant intervention effect on depression scores after controlling for baseline measures and demographic characteristics (β_TCP_=-0.075; SE = 0.036; *p* < 0.05), with intervention students displaying significantly fewer depressive symptoms than waitlist controls. Intervention effects on depressive symptoms were stronger for students from lower socioeconomic status backgrounds (β_Group*SES_ = 0.077, SE = 0.043, *p* < 0.05), and for transfer students (_βGroup*Transfer_ = − 0.189, SE = 0.091, *p* < 0.05).	After accounting for baseline loneliness and demographic characteristics, significant intervention effects were observed for students’ loneliness (β_TCP_ = − 0.074, SE = 0.035, *p* < 0.05), with intervention students displaying significantly less loneliness post intervention than waitlist controls. Intervention effects on loneliness were stronger for students who identified as a member of a minoritized racial or ethnic group, in comparison to White students (_βGroup*Minoritized_ = − 0.075, SE = 0.036, *p* < 0.05). A statistically significant intervention effect was reported on belongingness outcomes (β_TCP_ = 0.118, SE = 0.037, *p* < 0.01), and students who attended more sessions reported slightly greater gains in belongingness (β_attendance_ = 0.03, *p* < 0.05).
*The Connection Project*		
Risk of bias: Some concerns		
Haslam et al., 2016	There was a statistically significant decrease in depression outcomes in the intervention group four weeks post-intervention (T1_mean_ = 15.70; SD = 7.38, T2_mean_ = 13.00; SD = 10.37; *p* = 0.046), with 64.8% of participants experiencing improved depression outcomes. These changes were sustained six months post-intervention in the experimental group (T3-T1=-4.61; *p* = 0.026; ES = 0.47). Changes in depression scores between baseline and six months were not significant in the control group (T3-T1=-1.20; *p* = 0.634).	There was a statistically significant decrease in intervention participants’ loneliness scores (T1_meanloneliness_ = 2.83, SD = 0.38; T2_meanloneliness_ = 2.50, SD = 0.49; *p* < 0.001; d = 0.86) and social functioning impairment (T1_meansocial functioning_=2.29, SD = 0.52; T2_meansocial functioning_=2.15, SD = 0.56; *p* = 0.039) post-intervention, with 68.5% and 52.9% of intervention participants experiencing improved loneliness and social functioning outcomes, respectively. Follow-up data were not available for the control group.
*Groups 4 Health (G4H)*		
Risk of bias: Moderate		
Yoon et al., 2011	A statistically significant decrease in depression scores was reported in the intervention group (baseline_mean_=20.29, SD = 4.56; follow-up_mean_=15.26, SD = 6.14; t = 4.43, *p* < 0.001), but not in the control group (baseline_mean_=18.94, SD = 4.58; follow-up_mean_=17.88, SD = 6.52; t = 0.89, *p* = 0.390). A statistically significant difference was observed between intervention and control participants (mean-difference_intervention_=5.13, SD = 6.45; mean-difference_control_=1.06, SD = 6.84; t = 2.45, *p* = 0.017).	A statistically significant increase in interpersonal relationship score was reported in the intervention group (baseline_mean_=83.32; SD = 8.85; follow-up_mean_=88.71; SD = 10.07; t = 4.51; *p* < 0.001), but not in the control group (baseline_mean_=85.49; SD = 11.03; follow-up_mean_=84.82; SD = 11.09; t = 0.50; *p* = 0.619). A significant difference between intervention and control groups was reported for interpersonal relationships score changes (mean-difference_intervention_=5.39; SD = 6.65; Mean-difference_control_=0.67; SD = 7.62; t = 3.38; *p* = 0.001).
*Park’s interpersonal relationship program*		
Risk of bias: Serious		
**Online intervention with higher education students**		
Bruehlman-Senecal et al., 2020	There was no statistically significant intervention effect on depression scores overall (Intervention baseline_mean_=5.31, SD = 4.18, follow-up_mean_=5.71, SD = 4.14; Control baseline_mean_=6.65, SD = 5.52, follow-up_mean_=7.12, SD = 5.90; F < 1.60, *p* > 0.20). There was a statistically significant condition x baseline loneliness interaction to predict week four depression scores (F_1,209_ = 5.17, *p* = 0.02, ɳ_p_^2^ = 0.02). The Nod intervention buffered participants with higher baseline loneliness against heightened depression.	There was no evidence for an overall effect of intervention on loneliness (Intervention baseline_mean_ = 18.87, SD = 4.32, follow-up_mean_=16.71, SD = 4.73; Control baseline_mean_ = 18.91, SD = 4.40, follow-up_mean_ = 16.87, SD = 5.32; F_1,211_ = 0.05, *p* = 0.82; ηp^2^ = < 0.001). A statistically significant condition by baseline depression interaction on loneliness outcomes was reported (F_1,209_=9.65; *p* = 0.002; d = 0.40; 95%CI (0.15, 0.7)), suggesting that Nod buffered participants high in baseline depression from experiencing heightened loneliness. There was no evidence for an overall intervention effect on any of the three indices of college adjustment at week four, including social support, campus belonging, or social adjustment to college (all F values were < 1.40 and all *p*-values were > 0.23). A statistically significant condition by baseline loneliness interaction was reported to predict week four social support (F_1,210_ = 4.05, *p* = 0.045; ηp^2^ = 0.02) and campus belonging (F_1,209_ = 9.44, *p* = 0.002, η_p_^2^ = 0.04), suggesting that Nod buffered participants with higher baseline loneliness against reduced social support, campus belonging, and social adjustment at week 4.
*Nod app*		
Risk of bias: Some concerns		
**Intervention with youth involved in street life**		
McCay et al., 2011	No statistically significant changes in depression were reported for intervention (baseline_mean_=21.61, SD = 5.34, follow-up_mean_=17.95, SD = 4.14; t = 0.74, *p* = 0.48) or control (baseline_mean_=23.33, SD = 7.31, follow-up_mean_=23.67, SD = 13.35; t=-0.10, *p* = 0.93) groups.	A statistically significant improvement in social connectedness was reported in the intervention group post-intervention (baseline_mean_=78.67, SD = 16.68, follow-up_mean_=92.33, SD = 17.60, t=-2.28, df = 8, *p* = 0.05). No statistically significant change was reported for the comparison group.
*Relationship-based program*		
Risk of bias: Serious		

**Fig. 2:**
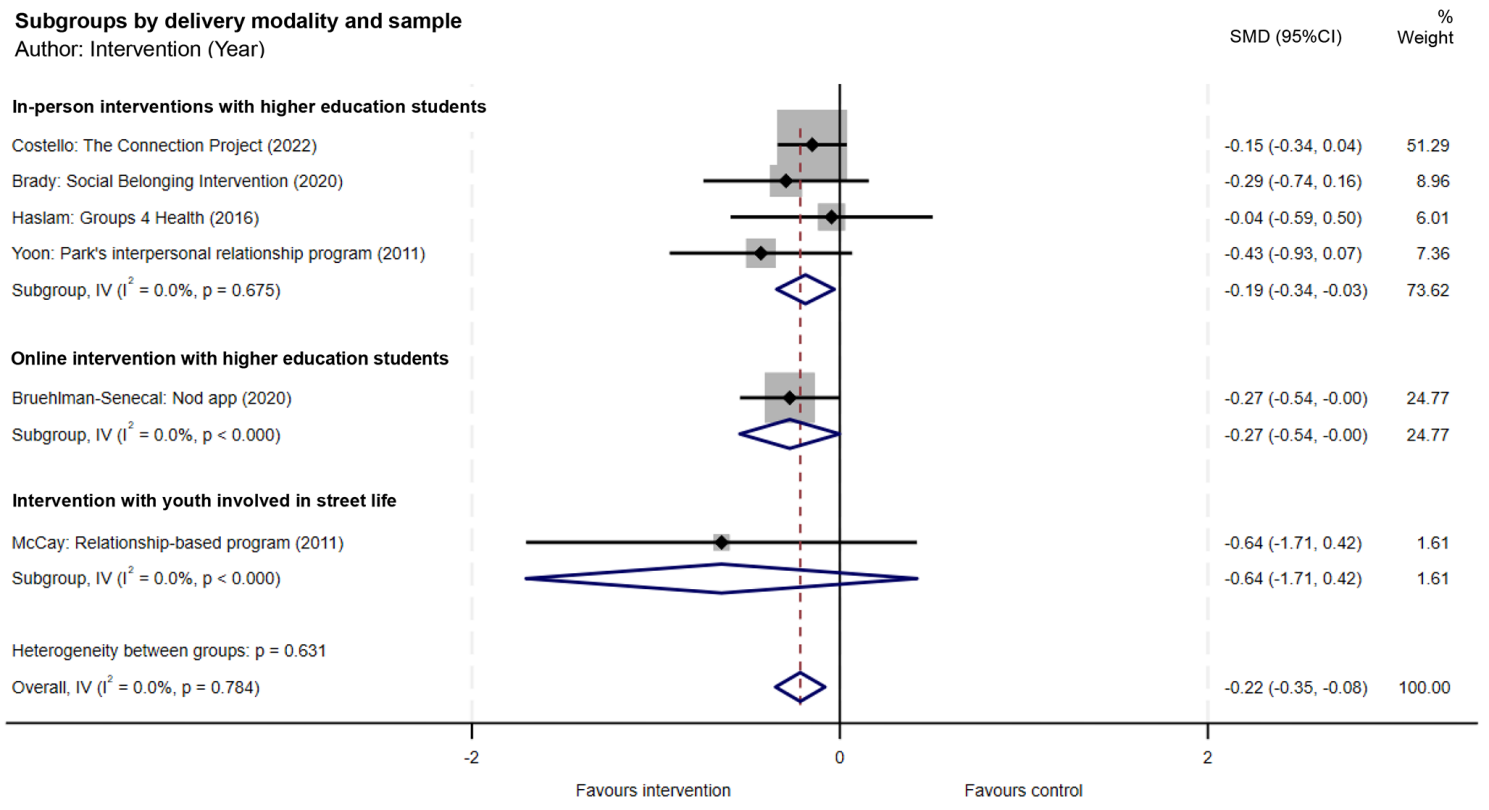
Forest plot of depression outcomes for all included social connection interventions with young adults (SMD = standardised mean difference; 95% CI = 95% confidence interval)

### Depression outcome

Overall, in random effects meta-analyses of all included studies, social connection interventions with young adults were associated with an overall mean reduction in depression scores (SMD = -0.22; 95% CI -0.35, -0.08; *p* = 0.002) relative to the control conditions. Heterogeneity was negligible (I^2^ = 0.0%, *p* = 0.784) (Fig. 2), but should be interpreted with caution due to the small number of studies [37]. Visual inspection of funnel plots did not indicate publication bias (see Supplementary File 4).

The size of the effect across the different intervention types (by delivery modality and sample) varied (Fig. 2). The four in-person interventions with higher education students [30, 32, 34, 35] were associated with a small overall mean reduction in depression scores (SMD = -0.19; 95% CI -0.34, -0.03; *p* = 0.020) relative to the control conditions. These interventions carried most weight in the meta-analysis (73.62%). Heterogeneity was negligible (I^2^ = 0.0%, *p* = 0.675), but should be interpreted with caution due to the small number of studies [37].

The one online intervention with higher education students, *Nod app*, was associated with a marginally significant reduction in depression scores (SMD = -0.27; 95% CI -0.54, -0.002; *p* = 0.048) relative to the control condition [31]. The one social connection intervention with youth involved in street life reported no statistically significant intervention effect on depression (SMD = -0.64; 95% CI -1.71, 0.42; *p* = 0.235) [33].

Sensitivity analyses were conducted; studies with serious risk of bias (one in-person intervention for higher education students [35] and the one intervention for youth involved in street life [33]) were excluded from the meta-analysis (see Supplementary File 5). Combined, the in-person and online interventions for higher education students were still associated with an overall mean reduction in depression scores relative to control conditions, but the effect was slightly reduced (SMD = -0.19; 95% CI -0.33, -0.05; *p* = 0.008). Heterogeneity was negligible (I^2^ = 0.0%, *p* = 0.802), but should be interpreted with caution due to the small number of studies [37]. When considering the in-person interventions for higher education students, removal of the serious risk of bias study reduced the overall mean intervention effect (SMD = -0.16, 95% CI -0.33, 0.01; *p* = 0.058).

#### Social connection and loneliness outcomes

The included studies reported several beneficial intervention effects for social connection outcomes, which were too heterogenous to pool. Of the in-person interventions with higher education students, intervention participants in the *Connection Project* [32] experienced improved post-intervention belongingness compared to control participants, with students who attended more sessions reporting slightly greater gains in belongingness. Compared to control participants, intervention participants experienced improved post-intervention social functioning in the *Groups 4 Health* program [34], improved interpersonal relationship scores in *Park’s interpersonal relationship program* [35], and improved social support for Black students in the *Social Belonging Intervention* [30].

The one online intervention with higher education students, *Nod app*, was the only intervention to have no effect on participants’ social connection outcomes overall. While interaction analyses revealed that the *Nod app* could buffer participants with high baseline loneliness against reduced social support and campus belonging at follow-up, these effects were small. The *Nod app* intervention showed low engagement and participants completed very few of the “social challenges” that were proposed in the app. In the one intervention with youth involved in street life [33], compared to control participants, intervention participants experienced improved post-intervention social connectedness.

**Fig. 3:**
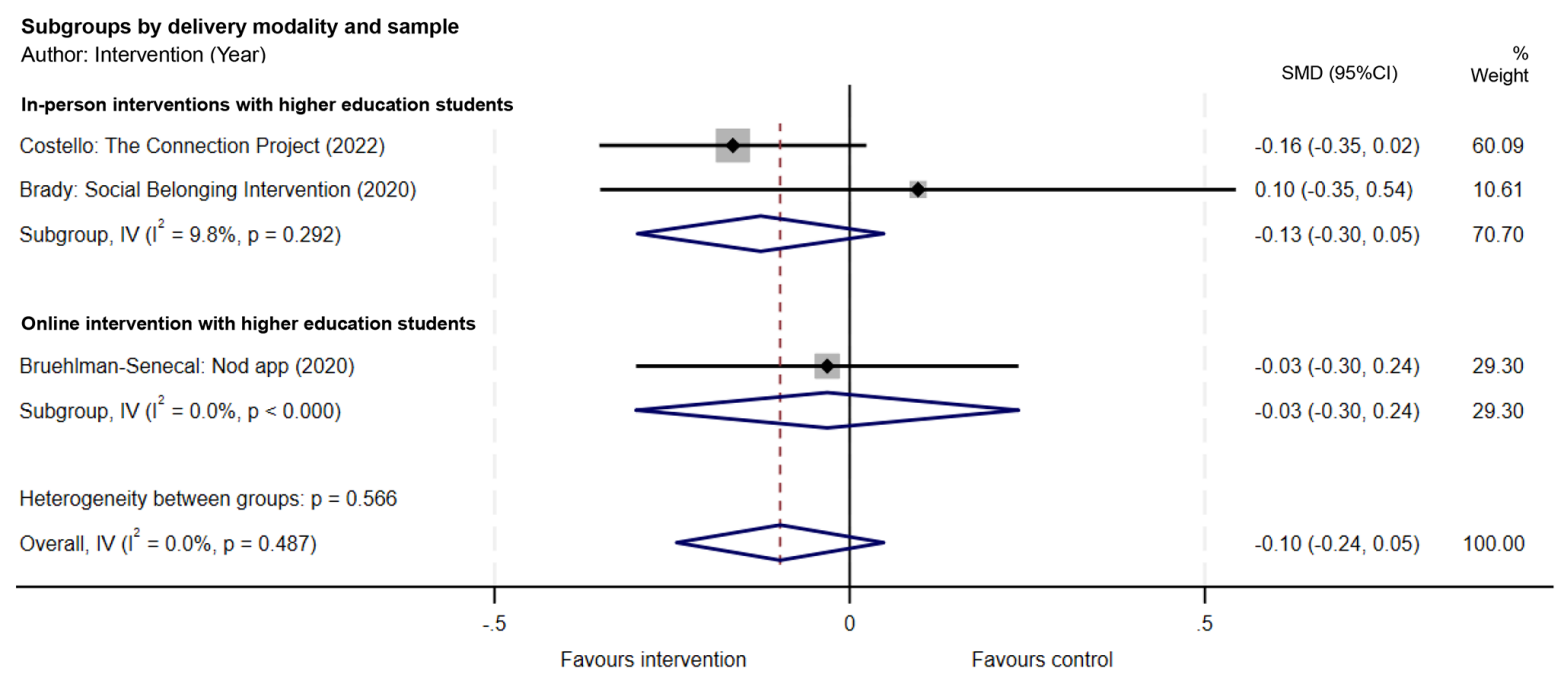
Forest plot of loneliness outcomes for social connection interventions with young adults (SMD = standardised mean difference; 95% CI = 95% confidence interval)

Four of the included studies measured loneliness as an outcome. Higher education students participating in the in-person *Group 4 Health* [34] program reported statistically significant decreases in loneliness post-intervention, but follow-up loneliness data were not available for the control group. Data from the other three studies measuring loneliness were pooled for meta-analysis [30–32]. Overall, in random effects meta-analyses, social connection interventions with young adults were not associated with an overall statistically significant mean reduction in loneliness scores (SMD = -0.10; 95% CI, -0.24, 0.05; *p* = 0.188) relative to the control conditions (Fig. 3). Heterogeneity was negligible (I^2^ = 0.0%, *p* = 0.487) (Fig. 3), but should be interpreted with caution due to the small number of studies [37]. A funnel plot was not produced due to the small number of studies. Sensitivity analyses were not conducted as none of the studies measuring loneliness had serious risk of bias.

## Discussion

This systematic review and meta-analysis aimed to identify and present interventions which address social connection/loneliness in young adults, and to describe the effectiveness of these interventions in changing depression outcomes and social connection/loneliness outcomes. To our knowledge, it is the first to examine the effectiveness of social connection interventions on depression and social connection/loneliness outcomes among non-clinical samples of young adults, aged 18–24 years. Prior to this review, little focus has been placed on young adults in the general population, who experience unique developmental challenges which make them especially vulnerable to both loneliness and depression [38]. This gap presents a missed public mental health opportunity, as social connection interventions may have the potential to reduce the overall mental illness burden in this age group through prevention of mental illness and promotion of wellbeing [39]. With three quarters of all lifetime cases of DSM-IV disorders reported to start by 24 years of age in the United States [40], early adulthood is an important life stage for targeted prevention interventions.

Only a small body of literature was identified, with six studies included in the review, emphasising a dearth of evidence in this area. Intervention effects on depression outcomes were mixed across the included studies, with three studies reporting beneficial effects and three reporting no, or limited, effects. However, pooled analysis indicated that, overall, social connection interventions appear to reduce depression for young adults. All studies reported some beneficial intervention effect for social connection outcomes. Importantly, none of the included studies explored whether any improvements in depression outcomes could be attributed to improvements in social connection or loneliness outcomes achieved through the intervention.

It is difficult to comment on which social connection interventions are likely to be most effective in reducing depression in young adults, due to the limited number of studies identified in the current review. Four of the six studies evaluated group-based social connection interventions [32–35], while two interventions took an individual approach through self-directed app-engagement [31] or a reading and reflection session [30]. The *Nod app* intervention showed low engagement and participants completed very few of the “social challenges” that were proposed in the app. This may highlight the importance of facilitating group-based interventions for social connection in young adults, as an alternative to placing the onus of engagement and social connection on individuals.

Evaluation of the *Nod app* intervention suggested that individuals with higher baseline depression and loneliness appeared to gain the greatest preventive benefits from the *Nod app* [31], which may indicate that app-based interventions could be a good starting point for individuals experiencing psychological and social difficulties. Similarly, in Haslam et al.’s *Group 4 Health* intervention [34], students who were experiencing moderate distress and social isolation at baseline made improvements through the intervention which were maintained at 6-months follow-up. This highlights the potential benefits of targeting social connection interventions to young adults known to be at mild-to-moderate risk of poor mental health and social isolation. In the *Connection Project*, intervention effects were strongest for young adults from lower socioeconomic status backgrounds or from minoritized racial or ethnic groups [32]. Similarly, only Black students in the *Social Belonging Intervention* experienced statistically significant gains in social support post-intervention [30]. This suggests that social connection interventions may be particularly important for young adults with characteristics which are known to be associated with an increased risk of social or mental health difficulties.

One of the most common contexts for delivering mental health promotion and prevention interventions is in school settings, as they are considered an ideal environment for implementation [20]. University settings could also offer a favourable setting for intervention implementation, which may explain why five of the studies included in the current review involved student samples in University / college settings. While many young adults attend University, early adulthood is also a period in which young people start working. Moving into potentially stressful and novel work environments can affect young adults’ mental health, and social support from supervisors and co-workers have been highlighted as important mitigating factors against job stress [41]. However, according to a recent Cigna survey of more than 6000 U.S. workers, 50% of young adult workers reported that they felt lonely at work and emotionally distant from their co-workers [42]. Other qualitative research has highlighted that young workers often feel invisible at work, have a poor sense of belonging to their employing organisation, and often experience relational deficiencies due to automation and individualisation of work practices [27]. With significant increases in working from home post-pandemic [43], there is a need to explore appropriate social connection interventions which can be applied for young working adults across different contexts.

Beyond educational and occupational settings, the social needs and mental health of young people who are not in education, employment, or training (NEET) should be considered. Findings from an epidemiological cohort study in England and Wales indicated that lonelier young adults were less confident in their employment prospects and were more likely to be out of work [44]. A recent systematic review and meta-analysis reported that NEET status was associated with a greater odds of mental ill-health (OR 1.28, CI 1.06–1.54) for young people [45], further highlighting the importance of targeted interventions for this group. Only one included study did not focus on a student population. In the study of youth involved in street life, no intervention effect on depression was reported, and the authors indicated that it was hard to engage this group in the intervention [33]. This group of young people experience a myriad of additional risk factors beyond social isolation, such as homelessness, neighbourhood safety issues, financial instability, or substance use, all of which have effects on both physical and mental health. Social interventions targeting high-risk groups need to consider these additional factors and the basic needs required for achieving health and safety. The current lack of social interventions for young people who are not in education, employment, or training, as well as the focus on young people in higher socioeconomic strata, is a limitation of the primary research available.

### Strengths and limitations of the review

This is the first review to examine the effectiveness of social connection interventions on depression outcomes among non-clinical samples of young adults and can serve as a useful resource for professionals working across a variety of sectors that directly and indirectly affect young peoples’ social connections and mental health. A robust methodology was followed in accordance with the AMSTAR-2 checklist.

It was beyond the scope of the current review to include grey literature, which may have led to publication bias or exclusion of studies that might be ongoing. This limitation was mitigated by contacting the corresponding author of each included study, requesting any additional studies. Qualitative research was also excluded from the review, which may limit our understanding of process factors and the acceptability of these interventions.

### Implications and future directions

This review highlights the potential mental health benefits of social connection interventions for some young adults, providing preliminary evidence for decision-makers wishing to address loneliness and depression in high-income higher education settings. Moving forward, more research is required to determine which social connection interventions are likely to be most effective in reducing depression in young adults across diverse settings. Specifically, future research should aim to (1) recruit larger samples, (2) have longer follow-up periods, (3) compare outcomes of online, in-person, self-directed, and group-based interventions, (4) consider how social connection interventions may serve young adults with characteristics associated with an increased risk of social or mental health difficulties, and (5) deliver social connection interventions to often-missed populations, such as young adults in the workplace, lower socioeconomic strata, or out of education, training, and employment.

## Conclusion

Social connection interventions show potential for increasing social connectedness in young adults, as well as effectiveness for reducing depressive symptoms. The evidence is currently limited to a relatively small effect from a few studies conducted primarily in high-income higher education settings. There is need for more high-quality social connection interventions and research for young adults across diverse settings in the general population.

## Electronic supplementary material

Below is the link to the electronic supplementary material.

Supplementary Material 1

## Data Availability

No datasets were generated or analysed during the current study.

## References

[1] Erskine H, Moffitt TE, Copeland W, Costello E, Ferrari A, Patton G et al (2015) A heavy burden on young minds: the global burden of mental and substance use disorders in children and youth. Psychol Med 45(7):1551–1563 25534496 10.1017/S0033291714002888 PMC5922255

[2] McGorry PD, Purcell R, Goldstone S, Amminger GP (2011) Age of onset and timing of treatment for mental and substance use disorders: implications for preventive intervention strategies and models of care. Curr Opin Psychiatry 24(4):301–306 21532481 10.1097/YCO.0b013e3283477a09

[3] Collishaw S (2015) Annual research review: secular trends in child and adolescent mental health. J Child Psychol Psychiatry 56(3):370–393 25496340 10.1111/jcpp.12372

[4] Bor W, Dean AJ, Najman J, Hayatbakhsh R (2014) Are child and adolescent mental health problems increasing in the 21st century? A systematic review. Australian New Z J Psychiatry 48(7):606–616 24829198 10.1177/0004867414533834

[5] Campion J (2018) Public mental health: key challenges and opportunities. BJPsych Int 15(3):51–54 31452534 10.1192/bji.2017.11 PMC6690256

[6] Cacioppo J, Cacioppo S (2018) The growing problem of loneliness. Lancet 391(10119):426 29407030 10.1016/S0140-6736(18)30142-9 PMC6530780

[7] McQuaid RJ, Cox SM, Ogunlana A, Jaworska N (2021) The burden of loneliness: implications of the social determinants of health during COVID-19. Psychiatry Res 296:113648 33348199 10.1016/j.psychres.2020.113648 PMC9754822

[8] Von Soest T, Luhmann M, Gerstorf D (2020) The development of loneliness through adolescence and young adulthood: its nature, correlates, and midlife outcomes. Dev Psychol 56(10):1919 32852969 10.1037/dev0001102

[9] Ding D, Eres R, Surkalim DL (2022) A lonely planet: time to tackle loneliness as a public health issue. BMJ 377:o1464 35700988 10.1136/bmj.o1464

[10] Victor CR, Yang K (2012) The prevalence of loneliness among adults: a case study of the United Kingdom. J Psychol 146(1–2):85–104 22303614 10.1080/00223980.2011.613875

[11] Hawkley LC, Buecker S, Kaiser T, Luhmann M (2022) Loneliness from young adulthood to old age: explaining age differences in loneliness. Int J Behav Dev 46(1):39–49 35001993 10.1177/0165025420971048 PMC8734589

[12] Buecker S, Mund M, Chwastek S, Sostmann M, Luhmann M (2021) Is loneliness in emerging adults increasing over time? A preregistered cross-temporal meta-analysis and systematic review. Psychol Bull 147(8):787 34898234 10.1037/bul0000332

[13] Groarke JM, Berry E, Graham-Wisener L, McKenna-Plumley PE, McGlinchey E, Armour C (2020) Loneliness in the UK during the COVID-19 pandemic: cross-sectional results from the COVID-19 psychological wellbeing study. PLoS ONE 15(9):e0239698 32970764 10.1371/journal.pone.0239698 PMC7513993

[14] McGinty EE, Presskreischer R, Han H, Barry CL (2020) Psychological distress and loneliness reported by US adults in 2018 and April 2020. JAMA 324(1):93–94 32492088 10.1001/jama.2020.9740 PMC7270868

[15] Rosenberg M, Luetke M, Hensel D, Kianersi S, Fu T-c, Herbenick D Depression and loneliness during COVID-19 restrictions in the United States, and their associations with frequency of social and sexual connections. medRxiv. 2020:2020.05.18.20101840. 10.1007/s00127-020-02002-8 PMC7778397 33386873

[16] Dawel A, Shou Y, Smithson M, Cherbuin N, Banfield M, Calear AL et al (2020) The effect of COVID-19 on mental health and wellbeing in a representative sample of Australian adults. Front Psychiatry 11:579985 33132940 10.3389/fpsyt.2020.579985 PMC7573356

[17] Lee CM, Cadigan JM, Rhew IC (2020) Increases in loneliness among young adults during the COVID-19 Pandemic and Association With increases in Mental Health problems. J Adolesc Health 67(5):714–717 33099414 10.1016/j.jadohealth.2020.08.009 PMC7576375

[18] Lund C, Brooke-Sumner C, Baingana F, Baron EC, Breuer E, Chandra P et al (2018) Social determinants of mental disorders and the Sustainable Development Goals: a systematic review of reviews. Lancet Psychiatry 5(4):357–369 29580610 10.1016/S2215-0366(18)30060-9

[19] Oswald TK, Nguyen MT, Mirza L, Lund C, Jones HG, Crowley G et al (2024) Interventions targeting social determinants of mental disorders and the sustainable development goals: a systematic review of reviews. Psychol Med 2024;54(8):1475–1499. 10.1017/S0033291724000333 38523245

[20] Filia K, Eastwood O, Herniman S, Badcock P (2021) Facilitating improvements in young people’s social relationships to prevent or treat depression: a review of empirically supported interventions. Translational Psychiatry 11(1):305 34021113 10.1038/s41398-021-01406-7 PMC8139977

[21] Eccles AM, Qualter P (2021) Alleviating loneliness in young people–a meta-analysis of interventions. Child Adolesc Mental Health 26(1):17–33 10.1111/camh.12389 32406165

[22] Kruzan KP, Williams KD, Meyerhoff J, Yoo DW, O’Dwyer LC, De Choudhury M et al (2022) Social media-based interventions for adolescent and young adult mental health: a scoping review. Internet Interventions 30:100578 10.1016/j.invent.2022.100578 PMC9530477 36204674

[23] Fakoya OA, McCorry NK, Donnelly M (2020) Loneliness and social isolation interventions for older adults: a scoping review of reviews. BMC Public Health 20:1–14 32054474 10.1186/s12889-020-8251-6 PMC7020371

[24] Beckers T, Maassen N, Koekkoek B, Tiemens B, Hutschemaekers G (2023) Can social support be improved in people with a severe mental illness? A systematic review and meta-analysis. Curr Psychol 42(17):14689–14699 10.1007/s12144-021-02694-4 PMC8802266 35125852

[25] Brooks H, Devereux-Fitzgerald A, Richmond L, Bee P, Lovell K, Caton N et al (2022) Assessing the effectiveness of social network interventions for adults with a diagnosis of mental health problems: a systematic review and narrative synthesis of impact. Soc Psychiatry Psychiatr Epidemiol 57(5):907–925 35138427 10.1007/s00127-022-02242-w PMC9042995

[26] Ma R, Mann F, Wang J, Lloyd-Evans B, Terhune J, Al-Shihabi A et al (2020) The effectiveness of interventions for reducing subjective and objective social isolation among people with mental health problems: a systematic review. Soc Psychiatry Psychiatr Epidemiol 55:839–876 31741017 10.1007/s00127-019-01800-z PMC7303071

[27] Wright SL, Silard AG (2022) Loneliness in Young Adult workers. Int J Environ Res Public Health 19(21):14462 36361344 10.3390/ijerph192114462 PMC9654605

[28] Kaye LK (2021) Exploring the socialness of social media. Computers Hum Behav Rep 3:100083

[29] Spineli LM, Pandis N (2021) Publication bias: graphical and statistical methods. Am J Orthod Dentofac Orthop 159(2):248–251 10.1016/j.ajodo.2020.11.005 33546830

[30] Brady ST, Cohen GL, Jarvis SN, Walton GM (2020) A brief social-belonging intervention in college improves adult outcomes for black americans. Sci Adv 6(18):eaay3689 32426471 10.1126/sciadv.aay3689 PMC7190359

[31] Bruehlman-Senecal E, Hook CJ, Pfeifer JH, FitzGerald C, Davis B, Delucchi KL et al (2020) Smartphone app to address loneliness among College students: pilot randomized controlled trial. JMIR Ment Health 7(10):e21496 33079071 10.2196/21496 PMC7609198

[32] Costello MA, Nagel AG, Hunt GL, Rivens AJ, Hazelwood OA, Pettit C et al (2022) Facilitating connection to enhance college student well-being: evaluation of an experiential group program. Am J Community Psychol 70(3–4):314–326 35575603 10.1002/ajcp.12601 PMC9666641

[33] McCay E, Quesnel S, Langley J, Beanlands H, Cooper L, Blidner R et al (2011) A relationship-based intervention to improve social connectedness in street-involved youth: a pilot study. J Child Adolesc Psychiatr Nurs 24(4):208–215 22044568 10.1111/j.1744-6171.2011.00301.x

[34] Haslam C, Cruwys T, Haslam SA, Dingle G, Chang MX (2016) Groups 4 health: evidence that a social-identity intervention that builds and strengthens social group membership improves mental health. J Affect Disord 194:188–195 26828756 10.1016/j.jad.2016.01.010

[35] Yoon HS, Kim GH, Kim J (2011) Effectiveness of an interpersonal relationship program on interpersonal relationships, self-esteem, and depression in nursing students. J Korean Acad Nurs 41(6):805–813 22310865 10.4040/jkan.2011.41.6.805

[36] Walton GM, Cohen GL (2011) A brief Social-Belonging Intervention Improves Academic and Health Outcomes of Minority Students. Science 331(6023):1447–1451 21415354 10.1126/science.1198364

[37] von Hippel PT (2015) The heterogeneity statistic I2 can be biased in small meta-analyses. BMC Med Res Methodol 15(1):35 25880989 10.1186/s12874-015-0024-z PMC4410499

[38] Arnett JJ (2000) Emerging adulthood: a theory of development from the late teens through the twenties. Am Psychol 55(5):469 10842426

[39] Pearce E, Myles-Hooton P, Johnson S, Hards E, Olsen S, Clisu D et al (2021) Loneliness as an active ingredient in preventing or alleviating youth anxiety and depression: a critical interpretative synthesis incorporating principles from rapid realist reviews. Translational Psychiatry 11(1):628 34893578 10.1038/s41398-021-01740-w PMC8661314

[40] Kessler RC, Berglund P, Demler O, Jin R, Merikangas KR, Walters EE (2005) Lifetime prevalence and age-of-onset distributions of DSM-IV disorders in the National Comorbidity Survey Replication. Arch Gen Psychiatry 62(6):593–602 15939837 10.1001/archpsyc.62.6.593

[41] Min JA, Lee CU, Lee C (2013) Mental health promotion and illness prevention: a challenge for psychiatrists. Psychiatry Investig 10(4):307–316 24474978 10.4306/pi.2013.10.4.307 PMC3902147

[42] Cigna (2020) Loneliness in the Workplace: 2020 U.S. Report

[43] Wax A, Deutsch C, Lindner C, Lindner SJ, Hopmeyer A (2022) Workplace loneliness: the benefits and detriments of Working from Home. Front Public Health 10:903975 10.3389/fpubh.2022.903975 PMC9184741 35692343

[44] Matthews T, Danese A, Caspi A, Fisher HL, Goldman-Mellor S, Kepa A et al (2019) Lonely young adults in modern Britain: findings from an epidemiological cohort study. Psychol Med 49(2):268–277 29684289 10.1017/S0033291718000788 PMC6076992

[45] Gariépy G, Danna SM, Hawke L, Henderson J, Iyer SN (2022) The mental health of young people who are not in education, employment, or training: a systematic review and meta-analysis. Soc Psychiatry Psychiatr Epidemiol 57(6):1107–1121 34931257 10.1007/s00127-021-02212-8 PMC8687877

