# Supplementary material for: Social connection interventions and depression in young adults: a systematic review and meta-analysis
Source: Soc Psychiatry Psychiatr Epidemiol. 2024 Aug 16;60(3):549–62. doi: 10.1007/s00127-024-02722-1 (PMC11870875; doi:10.1007/s00127-024-02722-1)
Supplement: Supplementary file 1 — Supplementary Material 1 [file 127_2024_2722_MOESM1_ESM.docx]

**Supplementary Files**

- Supplementary File 1 – PRISMA Checklist (page 2)
- Supplementary File 2 – Search strategies (page 8)
- Supplementary File 3 – Reasons for exclusion at full-text stage (page 11)
- Supplementary File 4 – Funnel plot for depression meta-analysis (page 17)
- Supplementary File 5 – Forest plot for depression sensitivity analysis excluding serious risk of bias studies (page 18)

**Supplementary File 1 – PRISMA Checklist**

| **Section and Topic** | **Item #** | **Checklist item** | **Location where item is reported** |
| --- | --- | --- | --- |
| **TITLE** | | |  |
| Title | 1 | Identify the report as a systematic review. | Title |
| **ABSTRACT** | | |  |
| Abstract | 2 | See the PRISMA 2020 for Abstracts checklist. | Abstract |
| **INTRODUCTION** | | |  |
| Rationale | 3 | Describe the rationale for the review in the context of existing knowledge. | Introduction section. |
| Objectives | 4 | Provide an explicit statement of the objective(s) or question(s) the review addresses. | Introduction section. |
| **METHODS** | | |  |
| Eligibility criteria | 5 | Specify the inclusion and exclusion criteria for the review and how studies were grouped for the syntheses. | Inclusion/ exclusion criteria sections. |
| Information sources | 6 | Specify all databases, registers, websites, organisations, reference lists and other sources searched or consulted to identify studies. Specify the date when each source was last searched or consulted. | Data source section. |
| Search strategy | 7 | Present the full search strategies for all databases, registers and websites, including any filters and limits used. | Supplementary File 2. |
| Selection process | 8 | Specify the methods used to decide whether a study met the inclusion criteria of the review, including how many reviewers screened each record and each report retrieved, whether they worked independently, and if applicable, details of automation tools used in the process. | Study screening and selection section. |
| Data collection process | 9 | Specify the methods used to collect data from reports, including how many reviewers collected data from each report, whether they worked independently, any processes for obtaining or confirming data from study investigators, and if applicable, details of automation tools used in the process. | Data extraction section. |
| Data items | 10a | List and define all outcomes for which data were sought. Specify whether all results that were compatible with each outcome domain in each study were sought (e.g. for all measures, time points, analyses), and if not, the methods used to decide which results to collect. | Data extraction section. |
|  | 10b | List and define all other variables for which data were sought (e.g. participant and intervention characteristics, funding sources). Describe any assumptions made about any missing or unclear information. | Data extraction section. |
| Study risk of bias assessment | 11 | Specify the methods used to assess risk of bias in the included studies, including details of the tool(s) used, how many reviewers assessed each study and whether they worked independently, and if applicable, details of automation tools used in the process. | Risk of bias assessments section. |
| Effect measures | 12 | Specify for each outcome the effect measure(s) (e.g. risk ratio, mean difference) used in the synthesis or presentation of results. | Statistical analysis section. |
| Synthesis methods | 13a | Describe the processes used to decide which studies were eligible for each synthesis (e.g. tabulating the study intervention characteristics and comparing against the planned groups for each synthesis (item #5)). | Narrative synthesis section. |
|  | 13b | Describe any methods required to prepare the data for presentation or synthesis, such as handling of missing summary statistics, or data conversions. | Narrative synthesis section. |
|  | 13c | Describe any methods used to tabulate or visually display results of individual studies and syntheses. | Narrative synthesis section. |
|  | 13d | Describe any methods used to synthesize results and provide a rationale for the choice(s). If meta-analysis was performed, describe the model(s), method(s) to identify the presence and extent of statistical heterogeneity, and software package(s) used. | Statistical analysis section. |
|  | 13e | Describe any methods used to explore possible causes of heterogeneity among study results (e.g. subgroup analysis, meta-regression). | Statistical analysis section. |
|  | 13f | Describe any sensitivity analyses conducted to assess robustness of the synthesized results. | Statistical analysis section. |
| Reporting bias assessment | 14 | Describe any methods used to assess risk of bias due to missing results in a synthesis (arising from reporting biases). | Statistical analysis section. |
| Certainty assessment | 15 | Describe any methods used to assess certainty (or confidence) in the body of evidence for an outcome. | Risk of bias assessments section. |
| **RESULTS** | | |  |
| Study selection | 16a | Describe the results of the search and selection process, from the number of records identified in the search to the number of studies included in the review, ideally using a flow diagram. | Results section. |
|  | 16b | Cite studies that might appear to meet the inclusion criteria, but which were excluded, and explain why they were excluded. | Results section and Supplementary File 3. |
| Study characteristics | 17 | Cite each included study and present its characteristics. | General study characteristics section and Table 1. |
| Risk of bias in studies | 18 | Present assessments of risk of bias for each included study. | Risk of bias of included studies section and Tables 2 and 3. |
| Results of individual studies | 19 | For all outcomes, present, for each study: (a) summary statistics for each group (where appropriate) and (b) an effect estimate and its precision (e.g. confidence/credible interval), ideally using structured tables or plots. | Main findings sections and Table 4. |
| Results of syntheses | 20a | For each synthesis, briefly summarise the characteristics and risk of bias among contributing studies. | General study characteristics section, Risk of bias of included studies section, and Main findings sections. |
|  | 20b | Present results of all statistical syntheses conducted. If meta-analysis was done, present for each the summary estimate and its precision (e.g. confidence/credible interval) and measures of statistical heterogeneity. If comparing groups, describe the direction of the effect. | Main findings sections. |
|  | 20c | Present results of all investigations of possible causes of heterogeneity among study results. | Main findings sections. |
|  | 20d | Present results of all sensitivity analyses conducted to assess the robustness of the synthesized results. | Main findings sections and Supplementary File 5. |
| Reporting biases | 21 | Present assessments of risk of bias due to missing results (arising from reporting biases) for each synthesis assessed. | NA |
| Certainty of evidence | 22 | Present assessments of certainty (or confidence) in the body of evidence for each outcome assessed. | Main findings sections. |
| **DISCUSSION** | | |  |
| Discussion | 23a | Provide a general interpretation of the results in the context of other evidence. | Discussion section. |
|  | 23b | Discuss any limitations of the evidence included in the review. | Discussion section. |
|  | 23c | Discuss any limitations of the review processes used. | Strengths and limitations of the review section. |
|  | 23d | Discuss implications of the results for practice, policy, and future research. | Discussion section and Implications and future directions section. |
| **OTHER INFORMATION** | | |  |
| Registration and protocol | 24a | Provide registration information for the review, including register name and registration number, or state that the review was not registered. | Methods section. |
|  | 24b | Indicate where the review protocol can be accessed, or state that a protocol was not prepared. | Methods section. |
|  | 24c | Describe and explain any amendments to information provided at registration or in the protocol. | Methods section. |
| Support | 25 | Describe sources of financial or non-financial support for the review, and the role of the funders or sponsors in the review. | Funding declaration section. |
| Competing interests | 26 | Declare any competing interests of review authors. | Competing interests section. |
| Availability of data, code and other materials | 27 | Report which of the following are publicly available and where they can be found: template data collection forms; data extracted from included studies; data used for all analyses; analytic code; any other materials used in the review. | NA |

*From:*  Page MJ, McKenzie JE, Bossuyt PM, Boutron I, Hoffmann TC, Mulrow CD, et al. The PRISMA 2020 statement: an updated guideline for reporting systematic reviews. BMJ 2021;372:n71. doi: 10.1136/bmj.n71

**Supplementary File 2 – Search strategies**

**PubMed (#1 AND #2 AND #3 AND #4) NOT #5**

| **#1 Population** | **#2 Intervention** | **#3 Outcome** | **#4 Study Type** | **NOT** | **Limits/Filters** |
| --- | --- | --- | --- | --- | --- |
| (“Young adult*” OR  “Graduate student*” OR  “College student*” OR  “University Student*” OR  “Emerging adult*” OR “young adult”[Mesh] OR “Education, Graduate”[Mesh] OR “postgraduate student*” OR “college graduate*” OR “university graduate*” OR “young people” OR “young person” OR youth) | ("Social Isolation"[Mesh] OR “social isolation” OR "Loneliness"[Mesh] OR “loneliness” OR "Social Interaction"[Mesh] OR “social interaction*” OR "Interpersonal Relations"[Mesh] OR “interpersonal relation*” OR "Social Networking"[Mesh] OR “social network*” OR "Online Social Networking"[Mesh] OR “online social network*” OR "Internet-Based Intervention"[Mesh] OR “internet-based intervention*” OR "Social Media"[Mesh] OR “social media” OR "Self-Help Groups"[Mesh] OR “Self-help group*” OR "Social Cohesion"[Mesh] OR “social cohesion” OR “social intervention*” OR “social connection” OR “networking” OR “support group*” OR “digital intervention*” OR “social relationship*” OR “in-person intervention*” OR “internet intervention*”) | ("Depression"[Mesh] OR "Depressive Disorder"[Mesh] OR “depressive disorder*” OR "Mental Health"[Mesh] OR “mental health” OR "Mood Disorders"[Mesh] OR depress* OR "mood disorder*" OR "affective disorder*") | ("Randomized Controlled Trial" [Publication Type] OR "Randomized Controlled Trials as Topic"[Mesh] OR "Controlled Clinical Trials as Topic"[Mesh] OR "Non-Randomized Controlled Trials as Topic"[Mesh] OR "Evaluation Studies as Topic"[Mesh] OR RCT OR “randomised controlled trial*” OR NRCT OR “non-randomised controlled trial*” OR “cluster randomised controlled trial*” OR “intervention stud*” OR “pre-post stud*” OR “prepost stud*” OR “pre-test post-test” OR “pre-post test” OR “evaluation stud*” OR pre-post study) | **("Review" [Publication Type] OR "Qualitative Research"[Mesh] OR "Books"[Mesh] OR "review" OR "qualitative" OR "review"[Title] OR “book” OR “chapter”)** | 1/1/2000-1/1/2023 |

**PsycInfo (#1 AND #2 AND #3 AND #4) NOT #5**

| **#1 Population** | **#2 Intervention** | **#3 Outcome** | **#4 Study Type** | **#5 NOT** | **Limits/Filters** |
| --- | --- | --- | --- | --- | --- |
| exp Young Adulthood/ OR Young Adulthood.mp. OR exp Emerging Adulthood/ OR Emerging Adulthood.mp. OR exp Students/ OR Student*.mp. OR exp College Graduates/ OR College Graduate*.mp. OR exp College Students/ OR College Student*.mp. OR exp Graduate Students/ OR Graduate Student*.mp. OR exp Postgraduate Students/ OR Postgraduate Student*.mp. OR university student*.mp. OR university graduate*.mp. OR young adult*.mp. OR young people.mp. OR young person.mp. OR youth.mp. | exp Social Isolation/ OR Social Isolation.mp. OR exp Loneliness/ OR Loneliness.mp. OR exp Social Interaction/ OR Social Interaction.mp. OR exp Interpersonal Interaction/ OR Interpersonal Interaction.mp. OR exp Interpersonal Relationships/ OR Interpersonal Relationship*.mp. OR exp Internet Social Networking/ OR Internet Social Networking.mp. OR exp Social Media/ OR Social Media.mp. OR exp Online Social Networks/ OR Online Social Network*.mp. OR Support Group*.mp. OR Social Cohesion.mp. OR exp Digital Intervention/ OR Digital Intervention*.mp. OR Interpersonal intervention*.mp. OR social networking.mp. OR internet-based intervention*.mp. OR internet intervention*.mp. OR self-help group*.mp. OR social intervention*.mp. OR social connection*.mp. OR networking.mp. OR social relationship*.mp. OR exp Web-Based Interventions/ OR Web-Based Intervention*.mp. | exp "Depression (Emotion)"/ OR "Depression (Emotion)".mp. OR Depressive disorder*.mp. OR exp Mental Health/ OR Mental Health.mp. OR exp Mood Disorders/ OR Mood Disorder*.mp. OR exp Affective Disorders/ OR Affective Disorder*.mp. OR depress*.mp. | exp Randomized Controlled Trials/ OR Randomi?ed Controlled Trial*.mp. OR "Randomi?ed Controlled Trial* as Topic".mp. OR controlled trial* without randomi?ation.mp. OR Evaluation Stud*.mp. OR Clinical Trial Publication Type .mp. OR Controlled Clinical Trial Publication Type .mp. OR RCT.mp. OR NRCT.mp. OR non-randomi?ed controlled trial*.mp. OR cluster randomi?ed controlled trial*.mp. OR intervention stud*.mp. OR pre-post stud*.mp. OR prepost stud*.mp. OR pre-test post-test.mp. OR pre-post test*.mp. | "Review (of Literature)" OR Systematic Review OR Literature Review OR Review Publication Type.mp. OR review.ti. OR Chapter.mp. OR Book*.mp. OR qualitative.mp. | 2020-2023 |

**Scopus (#1 AND #2 AND #3 AND #4) NOT #5**

| **#1 Population** | **#2 Intervention** | **#3 Outcome** | **#4 Study Type** | **#5 NOT** | **Limits/Filters** |
| --- | --- | --- | --- | --- | --- |
| TITLE-ABS-KEY {Young adults} OR “young adult” OR “young adulthood” OR  “Graduate students” OR {graduate students} OR  {College student} OR “college students”  OR {University Student} OR “university student” OR  “Emerging adult” OR “Education, Graduate” OR “emerging adulthood” OR “college graduate” OR “postgraduate student” OR “university graduate” OR “young people” OR {young people} OR “young person” OR youth | TITLE-ABS-KEY “Social Isolation” OR Loneliness OR {Social Interaction} OR “Social Interaction” OR “Interpersonal Interaction” OR “Interpersonal Relationship” OR “Internet Social Networking” OR “internet social network” OR “Social Media” OR “Online Social Networks” OR “online social networking” OR “Support Group” OR “Social Cohesion” OR “Digital Intervention” OR “Interpersonal intervention” OR “social networking” OR “internet-based intervention” OR “internet intervention” OR “self-help group” OR “social intervention” OR “social connection” OR “networking” OR “social relationship” OR “web-based intervention” | TITLE-ABS-KEY “Depression” OR {depression} OR “Depressive disorder” OR “depressive symptoms” OR “Mood Disorder” OR “Affective Disorder” | TITLE-ABS-KEY “Randomized Controlled Trial” OR “controlled trial without randomization” OR “Evaluation Study” OR “Controlled Clinical Trial” OR “RCT” OR “NRCT” OR “non-randomized controlled trial” OR “cluster randomised controlled trial” OR “intervention study” OR “pre-test post-test study” OR “pretest posttest study” OR “prepost study” OR “pre-post test” | TITLE-ABS-KEY {Systematic Review} OR {Literature Review} OR {Qualitative} OR {Chapter} OR {Book} OR {narrative review} OR {narrative synthesis} OR {realist review} | 2020-present |

**Supplementary File 3 – Reasons for exclusion at full-text stage**

Colour code: publication type/study design, age, clinical sample, outcome measure, full text missing

| **Citation** | **Decision + reason** |
| --- | --- |
| McCloskey, W., Iwanicki, S., Lauterbach, D., Giammittorio, D. M., & Maxwell, K. (2015). Are Facebook "Friends" Helpful? Development of a Facebook-Based Measure of Social Support and Examination of Relationships Among Depression, Quality of Life, and Social Support. *Cyberpsychol Behav Soc Netw*, *18*(9), 499-505. <https://doi.org/10.1089/cyber.2014.0538> | Exclude: wrong study type, development of a measure |
| Koenig Kellas, J., Horstman, H. K., Willer, E. K., & Carr, K. (2015). The benefits and risks of telling and listening to stories of difficulty over time: experimentally testing the expressive writing paradigm in the context of interpersonal communication between friends. *Health Commun*, *30*(9), 843-858. <https://doi.org/10.1080/10410236.2013.850017> | Exclude: wrong age group, wrong outcomes |
| Hsieh, N. L. (2010). A collaboration of student nurse coaches and students with mental illnesses in a college preparation project. *Psychiatr Rehabil J*, *33*(3), 200-206. <https://doi.org/10.2975/33.3.2010.200.206> | Exclude: clinical sample, no validated measures |
| Breland-Noble, A. M. (2012). Community and treatment engagement for depressed African American youth: The AAKOMA FLOA pilot [Empirical Study; Quantitative Study]. *Journal of Clinical Psychology in Medical Settings*, *19*(1), 41-48. [https://doi.org/https://dx.doi.org/10.1007/s10880-011-9281-0](https://doi.org/https:/dx.doi.org/10.1007/s10880-011-9281-0) | Exclude: adolescent + clinical population sample |
| Brock, R. L., O'Hara, M. W., & Segre, L. S. (2017). Depression treatment by non-mental-health providers: Incremental evidence for the effectiveness of listening visits [Clinical Trial; Empirical Study; Followup Study; Quantitative Study]. *American Journal of Community Psychology*, *59*(1-2), 172-183. [https://doi.org/https://dx.doi.org/10.1002/ajcp.12129](https://doi.org/https:/dx.doi.org/10.1002/ajcp.12129) | Exclude: wrong sample, wrong intervention |
| Cook, J. A., Copeland, M. E., Corey, L., Buffington, E., Jonikas, J. A., Curtis, L. C., Grey, D. D., & Nichols, W. H. (2010). Developing the evidence base for peer-led services: changes among participants following Wellness Recovery Action Planning (WRAP) education in two statewide initiatives. *Psychiatr Rehabil J*, *34*(2), 113-120. <https://doi.org/10.2975/34.2.2010.113.120> | Exclude: intervention type, outcome measures, population age |
| Hensel, D. J. (2022). Digital Interventions to Improve College and University Student Mental Health [Editorial]. Journal of Adolescent Health, 71(2), 141-142. <https://doi.org/10.1016/j.jadohealth.2022.05.017> | Exclude: editorial |
| Yu, S.-C. (2020). Does using social network sites reduce depression and promote happiness?: An example of Facebook-based positive interventions [Empirical Study; Quantitative Study]. International Journal of Technology and Human Interaction (IJTHI), 16(3), 56-69. [https://doi.org/https://dx.doi.org/10.4018/IJTHI.2020070104](https://doi.org/https:/dx.doi.org/10.4018/IJTHI.2020070104) | Exclude: wrong intervention type (positive intervention) does not aim to reduce loneliness |
| El Morr, C., Ritvo, P., Ahmad, F., & Moineddin, R. (2020). Effectiveness of an 8-week web-based mindfulness virtual community intervention for university students on symptoms of stress, anxiety, and depression: Randomized controlled trial [Article]. JMIR Mental Health, 7(7), Article e18595. <https://doi.org/10.2196/18595> | Exclude: wrong intervention type (mindfulness), no outcome for social connectedness |
| El Morr, C., Ritvo, P., Ahmad, F., & Moineddin, R. (2020). Effectiveness of an 8-week web-based mindfulness virtual community intervention for university students on symptoms of stress, anxiety, and depression: Randomized controlled trial [Article]. JMIR Mental Health, 7(7), Article e18595. <https://doi.org/10.2196/18595> | Exclude: wrong age group (18-65) + clinical sample |
| Bilge, A., & Engin, E. (2016). Effectiveness of the solution focused therapy which is based on interpersonal relationship theory: retrospective investigation [Empirical Study; Longitudinal Study; Retrospective Study; Quantitative Study; Treatment Outcome]. Anadolu Psikiyatri Dergisi, 17(4), 261-269. | Exclude: full paper not found + therapy + wrong study type |
| Rodriguez, L. M., Lee, K. D. M., Onufrak, J., Dell, J. B., Quist, M., Drake, H. P., & Bryan, J. (2020). Effects of a brief interpersonal conflict cognitive reappraisal intervention on improvements in access to emotion regulation strategies and depressive symptoms in college students. Psychol Health, 35(10), 1207-1227. <https://doi.org/10.1080/08870446.2019.1711090> | Exclude: intervention did not aim to increase connectedness, no measure for social connectedness |
| Klein, J. P., Berger, T., Schröder, J., Späth, C., Meyer, B., Caspar, F., Lutz, W., Arndt, A., Greiner, W., Gräfe, V., Hautzinger, M., Fuhr, K., Rose, M., Nolte, S., Löwe, B., Anderssoni, G., Vettorazzi, E., Moritz, S., & Hohagen, F. (2016). Effects of a Psychological Internet Intervention in the Treatment of Mild to Moderate Depressive Symptoms: Results of the EVIDENT Study, a Randomized Controlled Trial. Psychother Psychosom, 85(4), 218-228. <https://doi.org/10.1159/000445355> | Exclude: age (18-65), intervention did not aim at increasing social connection, CBT-based, no social connectedness outcome. |
| Davis, A. (2020). The effects of a web-based mindfulness meditation on stress and depressive symptoms in undergraduate pre-licensure nursing students: A pilot study *Dissertation Abstracts International Section A: Humanities and Social Sciences*, *81*(11-A), | Exclude: dissertation, no full text available |
| Lee, S., & Lee, E. (2020). Effects of Cognitive Behavioral Group Program for Mental Health Promotion of University Students. Int J Environ Res Public Health, 17(10). <https://doi.org/10.3390/ijerph17103500> | Exclude: cognitive behavioural intervention |
| Cho, W.-C., & Chen, C.-S. (2011). The effects of Gestalt group therapy on college experiences parental divorced in childhood [Empirical Study; Interview; Quantitative Study]. *Chinese Journal of Guidance and Counseling*, *30*, 69-100. | Exclude: no full text |
| Haddock, S. A., Weiler, L. M., Trump, L. J., & Henry, K. L. (2017). The Efficacy of Internal Family Systems Therapy in the Treatment of Depression Among Female College Students: A Pilot Study. J Marital Fam Ther, 43(1), 131-144. <https://doi.org/10.1111/jmft.12184> | Exclude: wrong intervention, no outcome measure of social connectedness |
| Bailey, E., Alvarez-Jimenez, M., Robinson, J., D'Alfonso, S., Nedeljkovic, M., Davey, C. G., Bendall, S., Gilbertson, T., Phillips, J., Bloom, L., Nicholls, L., Garland, N., Cagliarini, D., Phelan, M., McKechnie, B., Mitchell, J., Cooke, M., & Rice, S. M. (2020). An Enhanced Social Networking Intervention for Young People with Active Suicidal Ideation: Safety, Feasibility and Acceptability Outcomes. Int J Environ Res Public Health, 17(7). <https://doi.org/10.3390/ijerph17072435> | Exclude: Clinical population sample |
| Kanekar, A., Sharma, M., & Atri, A. (2009). Enhancing social support, hardiness, and acculturation to improve mental health among Asian Indian international students. Int Q Community Health Educ, 30(1), 55-68. <https://doi.org/10.2190/IQ.30.1.e> | Exclude: no outcome of depression + social connection |
| Byrom, N. (2018). An evaluation of a peer support intervention for student mental health [Article]. Journal of Mental Health, 27(3), 240-246. <https://doi.org/10.1080/09638237.2018.1437605> | Exclude: no social connection outcome |
| Li, T. M., Chau, M., Wong, P. W., Lai, E. S., & Yip, P. S. (2013). Evaluation of a Web-based social network electronic game in enhancing mental health literacy for young people. J Med Internet Res, 15(5), e80. <https://doi.org/10.2196/jmir.2316> | Exclude: aim was to assess mental health literacy. Wrong aim |
| Ashoorian, D., Albrecht, K. L., Baxter, C., Giftakis, E., Clifford, R., Greenwell-Barnden, J., & Wylde, T. (2019). Evaluation of Mental Health First Aid skills in an Australian university population. Early Interv Psychiatry, 13(5), 1121-1128. <https://doi.org/10.1111/eip.12742> | Exclude: cross sectional study + age group |
| O’Dea, B. (2021). Facilitating improvements in young people’s social relationships to prevent or treat depression: there’s an app for that [Letter]. Translational Psychiatry, 11(1), Article 470. <https://doi.org/10.1038/s41398-021-01597-z> | Exclude: letter, wrong article type |
| Sanatkar, S., Heinsch, M., Baldwin, P. A., Rubin, M., Geddes, J., Hunt, S., Baker, A. L., Woodcock, K., Lewin, T. J., Brady, K., Deady, M., Thornton, L., Teesson, M., & Kay-Lambkin, F. (2021). Factors predicting trial engagement, treatment satisfaction, and health-related quality of life during a web-based treatment and social networking trial for binge drinking and depression in young adults: Secondary analysis of a randomized controlled trial [Article]. JMIR Mental Health, 8(6), Article e23986. <https://doi.org/10.2196/23986> | Exclude: secondary analysis of a trial. |
| Ashton, L. M., Morgan, P. J., Hutchesson, M. J., Rollo, M. E., & Collins, C. E. (2017). Feasibility and preliminary efficacy of the 'HEYMAN' healthy lifestyle program for young men: a pilot randomised controlled trial. Nutr J, 16(1), 2. <https://doi.org/10.1186/s12937-017-0227-8> | Exclude: wrong study type, no outcome measures |
| Francis, S. E. B., Shawyer, F., Cayoun, B., Enticott, J., & Meadows, G. N. (2022). Group Mindfulness-Integrated Cognitive Behavior Therapy (MiCBT) Reduces Depression and Anxiety and Improves Flourishing in a Transdiagnostic Primary Care Sample Compared to Treatment-as-Usual: A Randomized Controlled Trial [Article]. Frontiers in Psychiatry, 13, Article 815170. <https://doi.org/10.3389/fpsyt.2022.815170> | Exclude: wrong age group and intervention |
| Fassnacht, D. B., Ali, K., van Agteren, J., Iasiello, M., Mavrangelos, T., Furber, G., & Kyrios, M. (2022). A Group-Facilitated, Internet-Based Intervention to Promote Mental Health and Well-Being in a Vulnerable Population of University Students: Randomized Controlled Trial of the Be Well Plan Program [Article]. JMIR Mental Health, 9(5), Article e37292. <https://doi.org/10.2196/37292> | Exclude: wrong age group, does not aim to increase social connection. |
| Haslam, C., Cruwys, T., Chang, M. X. L., Bentley, S. V., Alexander Haslam, S., Dingle, G. A., & Jetten, J. (2019). GROUPS 4 HEALTH reduces loneliness and social anxiety in adults with psychological distress: Findings from a randomized controlled trial [Article]. Journal of Consulting and Clinical Psychology, 87(9), 787-801. <https://doi.org/10.1037/ccp0000427> | Exclude: wrong age group |
| Haydon, M. D. (2022). Helping peers to promote well-being: A randomized controlled trial testing the benefits of an online prosocial intervention in young adult cancer survivors [Dissertation Clinical Trial; Empirical Study; Quantitative Study]. *Dissertation Abstracts International: Section B: The Sciences and Engineering*, *83*(3-B), | Exclude: no full text |
| Barber, C. R., & Weinberg, E. F. (2010). Integrative multidisciplinary treatment in the public sector: a pilot study. Bull Menninger Clin, 74(4), 263-282. <https://doi.org/10.1521/bumc.2010.74.4.263> | Exclude: Clinical sample |
| Sang, H., & Tan, D. (2018). Internalizing behavior disorders symptoms reduction by a social skills training program among chinese students: A randomized controlled trial [Article]. NeuroQuantology, 16(5), 104-109. <https://doi.org/10.14704/nq.2018.16.5.1312> | Exclude: age (9-12), suspected clinical problems |
| Aspy, D. J., & Proeve, M. (2017). Mindfulness and Loving-Kindness Meditation. Psychol Rep, 120(1), 102-117. <https://doi.org/10.1177/0033294116685867> | Exclude: no measure of depression, mindfulness intervention, not aiming to reduce loneliness |
| Rice, S., Gleeson, J., Davey, C., Hetrick, S., Parker, A., Lederman, R., Wadley, G., Murray, G., Herrman, H., Chambers, R., Russon, P., Miles, C., D'Alfonso, S., Thurley, M., Chinnery, G., Gilbertson, T., Eleftheriadis, D., Barlow, E., Cagliarini, D., . . . Alvarez-Jimenez, M. (2018). Moderated online social therapy for depression relapse prevention in young people: pilot study of a ‘next generation’ online intervention [Article]. Early Intervention in Psychiatry, 12(4), 613-625. <https://doi.org/10.1111/eip.12354> | Exclude: clinical population |
| Haug, S., Strauss, B., Gallas, C., & Kordy, H. (2008). New prospects for process research in group therapy: Text-based process variables in psychotherapeutic Internet chat groups. Psychother Res, 18(1), 88-96. <https://doi.org/10.1080/10503300701368008> | Exclude: clinical sample and age group |
| Farrer, L. M., Gulliver, A., Katruss, N., Fassnacht, D. B., Kyrios, M., & Batterham, P. J. (2019). A novel multi-component online intervention to improve the mental health of university students: Randomised controlled trial of the Uni Virtual Clinic. Internet Interv, 18, 100276. <https://doi.org/10.1016/j.invent.2019.100276> | Exclude: not aiming at increasing social connection and no measure of social connection |
| Alvarez-Jimenez, M., Rice, S., D'Alfonso, S., Leicester, S., Bendall, S., Pryor, I., Russon, P., McEnery, C., Santesteban-Echarri, O., Da Costa, G., Gilbertson, T., Valentine, L., Solves, L., Ratheesh, A., McGorry, P. D., & Gleeson, J. (2020). A Novel Multimodal Digital Service (Moderated Online Social Therapy+) for Help-Seeking Young People Experiencing Mental Ill-Health: Pilot Evaluation Within a National Youth E-Mental Health Service. J Med Internet Res, 22(8), e17155. <https://doi.org/10.2196/17155> | Exclude: wrong study aim, pilot evaluation. |
| Crisp, D., Griffiths, K., Mackinnon, A., Bennett, K., & Christensen, H. (2014). An online intervention for reducing depressive symptoms: Secondary benefits for self-esteem, empowerment and quality of life [Article]. Psychiatry Research, 216(1), 60-66. <https://doi.org/10.1016/j.psychres.2014.01.041> | Exclude: wrong age groups |
| DeAndrea, D., & Anthony, J. (2013). Online peer support for mental health problems in the United States: 2004-2010 [Empirical Study; Quantitative Study]. Psychological Medicine, 43(11), 2277-2288. [https://doi.org/https://dx.doi.org/10.1017/S0033291713000172](https://doi.org/https:/dx.doi.org/10.1017/S0033291713000172) | Exclude: not an intervention |
| Freeman, E., Barker, C., & Pistrang, N. (2008). Outcome of an online mutual support group for college students with psychological problems. Cyberpsychol Behav, 11(5), 591-593. <https://doi.org/10.1089/cpb.2007.0133> | Exclude: full text not found |
| Bautista, C. L., Ralston, A. L., Brock, R. L., & Hope, D. A. (2022). Peer coach support in internet-based cognitive behavioral therapy for college students with social anxiety disorder: Efficacy and acceptability [Clinical Trial; Empirical Study; Quantitative Study; Treatment Outcome]. Cogent Psychology Vol 9(1), 2022, ArtID 2040160, 9(1). [https://doi.org/https://dx.doi.org/10.1080/23311908.2022.2040160](https://doi.org/https:/dx.doi.org/10.1080/23311908.2022.2040160) | Exclude: Clinical population |
| Devilly, G. J., & Annab, R. (2008). A randomised controlled trial of group debriefing [Empirical Study; Quantitative Study]. Journal of Behavior Therapy and Experimental Psychiatry, 39(1), 42-56. [https://doi.org/https://dx.doi.org/10.1016/j.jbtep.2006.09.003](https://doi.org/https:/dx.doi.org/10.1016/j.jbtep.2006.09.003) | Exclude: wrong intervention type + no outcome measure for social connectedness |
| Adler, A. B., Williams, J., McGurk, D., Moss, A., & Bliese, P. D. (2015). Resilience training with soldiers during basic combat training: randomisation by platoon. Appl Psychol Health Well Being, 7(1), 85-107. <https://doi.org/10.1111/aphw.12040> | Exclude: wrong intervention type, age. |
| Holm, M., Tyssen, R., Stordal, K. I., & Haver, B. (2010). Self-development groups reduce medical school stress: a controlled intervention study. BMC Med Educ, 10, 23. <https://doi.org/10.1186/1472-6920-10-23> | Exclude: no measure of depression |
| Martínez-Hidalgo, M. N., Lorenzo-Sánchez, E., López García, J. J., & Regadera, J. J. (2018). Social contact as a strategy for self-stigma reduction in young adults and adolescents with mental health problems. Psychiatry Res, 260, 443-450. <https://doi.org/10.1016/j.psychres.2017.12.017> | Exclude: wrong intervention type, clinical population |
| Gater, R., Waheed, W., Husain, N., Tomenson, B., Aseem, S., & Creed, F. (2010). Social intervention for British Pakistani women with depression: Randomised controlled trial [Article]. British Journal of Psychiatry, 197(3), 227-233. <https://doi.org/10.1192/bjp.bp.109.066845> | Exclude: clinical population |
| Papadopoulos, K., Papakonstantinou, D., Montgomery, A., & Solomou, A. (2014). Social support and depression of adults with visual impairments. Res Dev Disabil, 35(7), 1734-1741. <https://doi.org/10.1016/j.ridd.2014.02.019> | Exclude: Age group and wrong article type |
| Hirani, S. S., Norris, C. M., Van Vliet, K., Van Zanten, S. V., Karmaliani, R., & Lasiuk, G. (2018). Social support intervention to promote resilience and quality of life in women living in Karachi, Pakistan: A randomized controlled trial [Clinical Trial; Empirical Study; Quantitative Study]. International Journal of Public Health, 63(6), 693-702. [https://doi.org/https://dx.doi.org/10.1007/s00038-018-1101-y](https://doi.org/https:/dx.doi.org/10.1007/s00038-018-1101-y) | Exclude: outcome measures and age group |
| Vincke, J., & van Heeringen, K. (2004). Summer holiday camps for gay and lesbian young adults: an evaluation of their impact on social support and mental well-being. J Homosex, 47(2), 33-46. <https://doi.org/10.1300/J082v47n02_02> | Exclude: age group and study design |
| Boschloo, L., Cuijpers, P., Karyotaki, E., Berger, T., Moritz, S., Meyer, B., & Klein, J. P. (2019). Symptom-specific effectiveness of an internet-based intervention in the treatment of mild to moderate depressive symptomatology: The potential of network estimation techniques [Article]. Behaviour Research and Therapy, 122, Article 103440. <https://doi.org/10.1016/j.brat.2019.103440> | Exclude: age - adults |
| Hatch, S., Roddy, M. K., Doss, B. D., Rogge, R. D., Esplin, C. R., & Braithwaite, S. R. (2020). Texts 4 romantic relationships-A randomized controlled trial [Clinical Trial; Empirical Study; Quantitative Study]. Journal of Couple & Relationship Therapy, 19(2), 115-135. [https://doi.org/https://dx.doi.org/10.1080/15332691.2019.1667936](https://doi.org/https:/dx.doi.org/10.1080/15332691.2019.1667936) | Exclude: no measure of depression |
| Klein, J. P., Spath, C., Schroder, J., Meyer, B., Greiner, W., Hautzinger, M., Lutz, W., Rose, M., Vettorazzi, E., Andersson, G., Hohagen, F., Moritz, S., & Berger, T. (2017). Time to remission from mild to moderate depressive symptoms: One year results from the EVIDENT-study, an RCT of an internet intervention for depression [Clinical Trial; Empirical Study; Quantitative Study; Treatment Outcome]. Behaviour Research and Therapy, 97, 154-162. [https://doi.org/https://dx.doi.org/10.1016/j.brat.2017.07.013](https://doi.org/https:/dx.doi.org/10.1016/j.brat.2017.07.013) | Exclude: age, wrong aim no measure of social connection |
| Yu, S. C., Sheldon, K. M., Lan, W. P., & Chen, J. H. (2020). Using social network sites to boost savoring: Positive effects on positive emotions [Article]. International Journal of Environmental Research and Public Health, 17(17), 1-11, Article 6407. <https://doi.org/10.3390/ijerph17176407> | Exclude: wrong aim and no measure of social connection |
| Bantjes, J., Kazdin, A. E., Cuijpers, P., Breet, E., Dunn-Coetzee, M., Davids, C., Stein, D. J., & Kessler, R. C. (2021). A web-based group cognitive behavioral therapy intervention for symptoms of anxiety and depression among university students: Open-label, pragmatic trial [Article]. JMIR Mental Health, 8(5), Article e27400. <https://doi.org/10.2196/27400> | Exclude: age and no social connection measure |
| Hu, M. (2009). Will online chat help alleviate mood loneliness? Cyberpsychol Behav, 12(2), 219-223. <https://doi.org/10.1089/cpb.2008.0134> | Exclude: full text not found |

**Supplementary File 4 – Funnel plot for depression meta-analysis**

**Supplementary File 5 – Forest plot for depression sensitivity analysis, excluding serious risk of bias studies**

**Subgroups by delivery modality and sample**

Author: Intervention (Year)

%

Weight

SMD (95%CI)


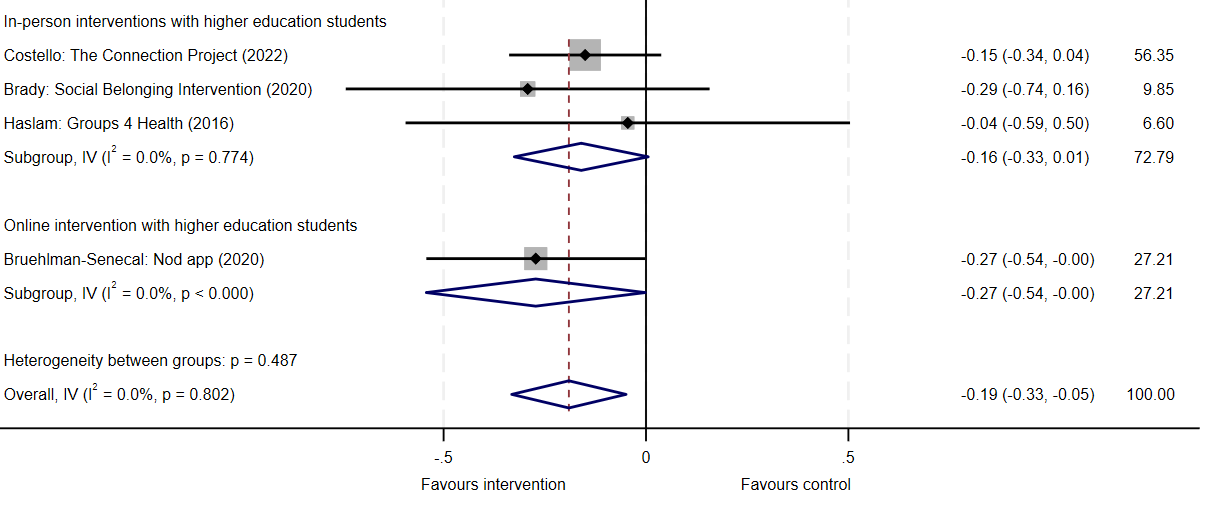


**Online intervention with higher education students**

**In-person interventions with higher education students**
